# Predictive value of NLR and PLR in missed miscarriage

**DOI:** 10.1002/jcla.24250

**Published:** 2022-02-01

**Authors:** Dan Liu, Xinyan Huang, Zhengxian Xu, Minzhi Chen, Manyu Wu

**Affiliations:** ^1^ Department of Women's HealthCare Affiliated Foshan Women and Children Hospital Southern Medical University Foshan China

**Keywords:** mean platelet volume, missed miscarriage, neutrophil to lymphocyte ratio, platelet to lymphocyte ratio

## Abstract

**Background:**

The aim of the study was to investigate the predictive value of neutrophil to lymphocyte ratio (NLR) and platelet to lymphocyte ratio (PLR) in missed miscarriage.

**Methods:**

In this retrospective cohort study, a total of 400 women (involving 200 with missed early miscarriage and 200 with normal pregnancy but terminate by artificial abortion) were included. General clinical data and complete blood count (CBC) such as white blood cells (WBC), red blood cells (RBC), platelet (PLT), red blood cell distribution width‐standard deviation (RDW‐SD), platelet distribution width (PDW), mean platelet volume (MPV), neutrophil count, and lymphocyte count were collected, and the NLR and PLR were calculated for both groups. Receiver operating characteristic curve (ROC) was used to calculate the predictive value.

**Results:**

There was no significant difference in the WBC, RBC, PLT, RDW‐SD, PDW, neutrophil, lymphocyte, NLR, and PLR between the two groups (*p* > 0.05).But MPV was lower in the missed early miscarriage group than in the control group (*p* < 0.05), and the area under the working curve (AUC) of ROC was 0.58, specificity and sensitivity was 69% and 47%, respectively.

**Conclusion:**

NLR and PLR were not the suitable indictor for missed miscarriage, but MPV should be a concern in the first trimester.

## Graphical abstract

In our study, WBC, RBC, PLT, RDW‐SD, PDW, neutrophil, lymphocyte, NLR, and PLR were similar between the two groups (p ＞ 0.05). But MPV value was lower in the missed miscarriage group than in the controls (p ＜ 0.05). (Table 2) We found that NLR and PLR values do not have any determining effect on the presence of missed miscarriage but reminds clinicians to pay attention to MPV, the rapid and simple parameters in early pregnancy patients.

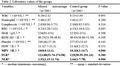

## 1 INTRODUCTION

Pregnancy failure is a common complication of pregnancy. Missed miscarriage is one of the various presentations, pathological manifestation by the embryo or fetus has died but remains in the uterus within 20 weeks of gestation.^1^ Complications such as uterine adhesion, secondary infertility, bleeding, and infection after missed miscarriage seriously endanger women's physical and mental health. At present, the incidence of missed miscarriage showed a rising trend and occurs in about 8%–20% of clinically diagnosed pregnancies.^2^, ^3^ Presently, multiple etiologic factors including parental genetic factors, immunological factors, endocrine disorders, uterine abnormalities, thrombophilia, infections, and environmental factors have been identified for missed miscarriage. However, the exact pathophysiologic mechanism remains unclear.

Nowadays, growing evidence indicates the significant role played by immunological and inflammatory factors in the development of missed miscarriage. During human pregnancy, a semi‐allogeneic fetus implants into the endometrium.^4^ Maternal systemic inflammatory processes can take place due to a sterile maternal immune reaction against allo‐antigens on the fetus or trophoblast.^5^ Many inflammation‐related serum markers, such as periostin, Th‐1, TNF‐α, and Th‐17 are associated with miscarriage and influence the circulating inflammatory condition.^6^, ^7^ But these are uneasily available, expensive, and not routinely tested.

Complete blood count (CBC) parameters have been regarded as a rapid and simple parameters indicative of systemic inflammation and stress. NLR and PLR are ratio indices calculated by inflammatory activators (neutrophils/platelets) and inflammatory regulators (lymphocytes) that are considered effective indicators of systemic inflammation and immune balance. NLR was introduced as a marker of underlying inflammatory burden in various diseases including irritable bowel syndrome,^8^ type 2 DM,^9^ thyroiditis,^10^ and inflammatory bowel disease.^11^ The predictive role of PLR has been reported in diabetes mellitus,^12^ thyroid conditions,^13^ malignancy,^14^ and liver fibrosis.^15^ Additionally, MPV is a surrogate marker of platelet activation^16^ and found to be associated with inflammatory conditions such as rheumatoid arthritis,^17^ cardiac conditions,^18^ vertebral discopathies,^19^ diabetes,^20^ obesity,^21^ nasal polyps,^22^ and malignancy.^23^

However, little work has been done on these simple and routine blood examination and missed miscarriage. Therefore, in the present study, we aimed to evaluate whether the CBC parameters, especially NLR and PLR, would be useful serum markers in predicate of missed miscarriage.

## 2 MATERIALS AND METHODS

### 2.1 Subjects

This retrospective comparative study was conducted at Affiliated Foshan Maternity & Child Healthcare Hospital, Southern Medical University. The study was approved by the ethics committee of Affiliated Foshan Maternity & Child Healthcare Hospital, Southern Medical University.

The study used data that were gathered from January 2018 to December 2020, women aging between 18 and 35 years old, gestational age ≤12 weeks, singleton pregnancy. The inclusion criteria of the study group were missed miscarriage patients, who diagnosed by ultrasonic, such as the length of head and hip was ≥7 mm, and no fetal heartbeat was found; the average diameter of pregnancy sac in uterine cavity was ≥25 mm, and no embryo was found; No yolk sac was found in intrauterine pregnancy, and no embryo and fetal heartbeat were found after 2 weeks; yolk sac could be seen in intrauterine pregnancy, and there was still no fetal heartbeat after 11 days.^24^, ^25^ The inclusion criteria of the control group were pregnant women with normal pregnancy who terminate their unwanted pregnancy by artificial abortion. The following patients were excluded: patients with inadequate data, chromosomal abnormality, uterine structural abnormalities, a history of recurrent miscarriage, acute or chronic infectious diseases, cancer, patients receiving progesterone therapy, any other medical condition needing drug treatment, and smoking during pregnancy.

### 2.2 Sample collection

Age, gestational week, gravida, parity, body weight, and height were obtained by examining the medical records of patients. The gestational week was determined by sonographic measurement. CBC parameters including white blood cells (WBC), red blood cells (RBC), platelet (PLT), red blood cell distribution width‐standard deviation (RDW‐SD), platelet distribution width (PDW), mean platelet volume (MPV), neutrophil count, and lymphocyte count were determined. Neutrophil to lymphocyte ratio (NLR) was defined by dividing the neutrophil count by lymphocyte count, and platelet to lymphocyte ratio (PLR) was defined as platelet count divided by lymphocyte count. Preoperative CBC parameters were compared between the missed miscarriage and control groups.

### 2.3 Statistical analysis

Statistical analyses were performed using SPSS windows version 20.0 software (SPSS Inc.). The Kolmogorov–Smirnov normality test was run for checking the distribution of CBC, and the Levene statistic test was used to test the homogeneity of variances. Unpaired Student's *t* test were conducted for comparison of normally distributed variables. The results of normally distributed variables are presented as the mean ± standard deviation (SD). Mann–Whitney *U* test was used for comparison of non‐normally distrusted hematological parameters, and the results were expressed as median (minimum‐maximum) values. A *p* value <0.05 was considered statistically significant. Receiver operating characteristic curves (ROC) were used to evaluate the predictive value of MPV in missed early miscarriage predict, and area under the curve (AUC) determined the discriminative ability of MPV.

## 3 RESULTS

A total of 200 patients with missed miscarriage was compared with 200 participants in the control group who had normal pregnancies. The two groups were similar in terms of maternal age, gestational age, BMI, gravidity, and parity (*p* > 0.05). (Table 1).

**TABLE 1:** Demographic and clinical features of the groups

Variables	Missed miscarriage(*n* = 200)	Control group(*n* = 200)	*p* value
Age(years)*	28(18–34)	27(18–34)	0.538
BMI(kg/m^2^)**	22.14 ± 2.69	21.96 ± 2.15	0.187
gestational weeks*	7(6–12)	7(6–12)	0.230
Gravida*	2(1–6)	1(1–5)	0.482
Parity*	1(0–3)	1(0–4)	0.425

Note

* — Median (minimum‐maximum).

** — Mean ± standard deviation.

WBC, RBC, PLT, RDW‐SD, PDW, neutrophil, lymphocyte, NLR, and PLR were similar between the groups (*p* > 0.05). MPV value was lower in the missed miscarriage group than in the control group (*p* < 0.05). (Table 2).

**TABLE 2:** Laboratory values of the groups

Variables	Missed miscarriage(*n* = 200)	Control group(*n* = 200)	*p* value
WBC(×10^9/L)**	8.18 ± 2.12	8.13 ± 1.94	0.091
Neutrophil(×10^9/L)**	5.48 ± 1.87	5.66 ± 1.67	0.290
Lymphocyte(×10^9/L)*	2.03(0.96–3.75)	2.10(0.82–3.85)	0.316
RBC(×10^12/L)*	4.25(3.20–7.63)	4.25(3.18–6.22)	0.685
HGB(g/L)*	126(91–151)	127(91–151)	0.308
RDW‐SD(fL)*	40.25(31.90–48.8)	40.45(30.80–51.50)	0.368
Platelet(×10^9/L)**	249.68 ± 57.50	257.87 ± 53.65	0.141
PDW(fL)*	11.1(7.7–21.5)	11.4(8.2–22.5)	0.351
MPV(fL)*	10(8.0–13.1)	10.3(8.3–14.7)	0.006
PLR*	123.88(51.74–274.58)	125(58.55–291.24)	0.380
NLR*	2.52(1.15–11.74)	2.66(1–7.78)	0.906

Note

* — median (minimum‐maximum).

** — mean ± standard deviation.

**FIGURE 1:**
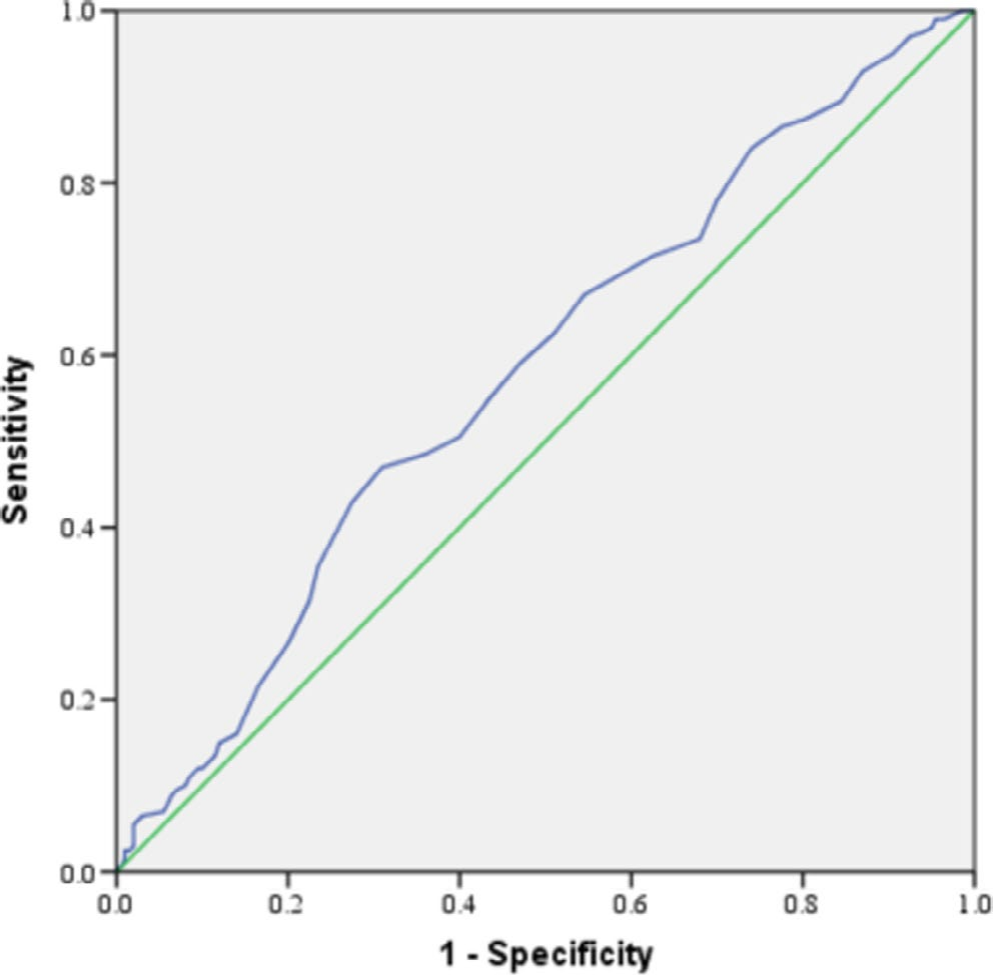
ROC curve analysis for MPV

Further analysis showed that the MPV value showed that the AUC of ROC was 0.58, the cutoff value is 10.45, and the specificity and sensitivity was 69% and 47%, respectively. (Figure 1).

## 4 DISCUSSION

In this study, we found that WBC, RBC, PLT, RDW‐SD, PDW, neutrophil, lymphocyte, NLR, and PLR were similar in missed miscarriage group and the control group (*p* > 0.05). Interestingly, MPV was found to be lower in the missed miscarriage group than in the controls (*p* < 0.05). Further analysis showed that the best predicted values for missed miscarriage based on the AUC was MPV with the optimal cutoff value of 10.45, but the AUC was 0.58, the specificity and sensitivity was 69% and 47%, respectively.

Inflammation is essential for successful female reproduction.^6^, ^26^ At different stages of pregnancy, the maternal immune system presents different inflammatory states.^27^ In the beginning, a moderate inflammatory environment is conducive to embryo implantation. Then, the local decidua needs to establish an anti‐inflammatory and immune‐tolerant microenvironment to ensure the survival and growth of the embryo. At the time of delivery, the microenvironment of the decidua shifts toward the proinflammatory direction again. An excessive inflammatory reaction has been associated with miscarriage or other pregnancy complications such as pre‐eclampsia or premature labor.^28^

In recent years, as systemic markers of inflammation, with advantages of convenience, simplicity, sensitivity, versatility and speed, and CBC parameters have attracted more and more attention in miscarriage. NLR and PLR were the most commonly used inflammation markers among CBC parameters. However, the relationship between NLR, PLR and miscarriage are confusing. Oglak et al.^29^ found that NLR and PLR values were significantly higher in the early pregnancy loss group than the control group who had given birth at term, while Yakıştıran et al.^30^ found that PLR and NLR levels were decreased in miscarriages compare to elective abortion and healthy group. Additionally, Bas et al.^31^ show that NLR was positive predictive markers, and PLR was a negative predictive marker for the evaluation of spontaneous abortion, while PLR was higher in miscarriage group, NLR was no statistically significant differences compared with the control group in another research.^32^ Unlike those studies above, Gorkem et al.^33^ reported that there was no significant association between healthy pregnant women, threatened abortion group and the spontaneous abortion group in NLR and PLR, and Christoforaki et al.^34^ also reported that NLR and PLR values were not significantly different between the live birth group and miscarriage group. These were consistent with our research in miscarriage.

As for missed miscarriage, the relevant research is limited. To our knowledge, only two studies research on NLR and PLR in missed miscarriage. Biyik et al.^35^ showed that NLR and PLR values were higher in the missed miscarriage group than in the healthy pregnant women group. Kim et al.^36^ showed that NLR was the prognostic factor for distinguishing between the missed abortion and threatened abortion. In contrast to these studies, we found that the NLR and PLR values were similar in missed miscarriage and normal pregnancy group (*p* > 0.05). This inconsistent fact is most probably due to the differences between the methods and/or equipment used in the missed miscarriage women. Meanwhile, all of this inflammation in fete‐maternal interface is similar to systemic or not still need further research.

To our surprise, we observed that MPV was lower in the missed miscarriage group than in the control. As far as we know, the only research Biyik et al.^35^ has similar results with ours. These may be the cause of inflammation. According to our searches, increased MPV levels are observed in low‐grade inflammatory diseases, while decreased MPV levels are observed in high‐grade inflammatory diseases.^37^, ^38^ Increased MPV was observed in cardiovascular diseases, cerebral stroke, respiratory diseases, chronic renal failure, intestine diseases, rheumatoid diseases, diabetes, and various cancers, decreased MPV was noted in tuberculosis during disease exacerbation, ulcerative colitis, SLE in adult, and different neoplastic diseases.^39^ There are many studies have reported the relationship between miscarriage and MPV, but findings are also still confusing. Study show that MPV was significantly lower in miscarriage than the women given birth at term without complication and healthy control patients.^32^ However, there are also studies found that MPV levels was higher or no difference in the miscarriage group vs the control group. Confounding factors can be a probable reason for the different findings observed among the studies, and the pathophysiology between miscarriage and missed miscarriage is different, more researches are needed to illustrate the problem.

The limitations of this study include the fact that, firstly, the embryos of the control group were alive at the time of termination of pregnancy, but the subsequent pregnancy was unknown, which may cause bias in the data. Secondly, the missed miscarriage CBC parameters was detected when women confirm a missed early miscarriage, and we do not know whether CBC parameters abnormalities will only occur after the missed miscarriage, it still needs a systematic and detailed follow‐up of the CBC parameters in missed early miscarriage. Third, this study only included a single center of data.

## 5 CONCLUSION

In our study, we found that NLR and PLR values do not have any determining effect on the presence of missed miscarriage. MPV was found to be lower in the missed miscarriage group than in the controls, although it was not powerful enough to predict of missed miscarriage, but reminds clinicians to pay attention to the rapid and simple parameters in early pregnancy patients. In a word, further large‐sample, multicenter and systematic prospective studies are needed.

## CONFLICT OF INTEREST

The authors declare that they have no conflict of interest.

## AUTHOR CONTRIBUTIONS

Manyu Wu contributed to study concept and design. Dan Liu and Manyu Wu wrote the article. Dan Liu, Xinyan Huang, Zhengxian Xu, and Minzhi Chen collected the samples. Xinyan Huang, Zhengxian Xu, and Minzhi Chen performed the statistical analysis. All authors commented on previous versions of the article. All authors read and approved the final article.

## Data Availability

The data that support the findings of this study are available from the corresponding author upon reasonable request.
